# The Role of Rarely Studied Chemokines in Tumor Progression in Multiple Myeloma (MM)

**DOI:** 10.3390/cancers18040673

**Published:** 2026-02-18

**Authors:** Jan Korbecki, Mateusz Bosiacki, Rafał Stelmach, Katarzyna Barczak

**Affiliations:** 1 Institute of Health Sciences, Collegium Medicum, University of Zielona Góra, 28 Zyty St., 65-046 Zielona Góra, Poland; jan.korbecki@onet.eu; 2 Department of Biochemistry and Medical Chemistry, Pomeranian Medical University in Szczecin, 72 Powstańców Wlkp. Av., 70-111 Szczecin, Poland; 3 Department of Oral Surgery, Central Clinical Hospital, Medical University of Lodz, 92-213 Łódź, Poland; rafal.stelmach@umed.lodz.pl; 4 Department of Endodontic Surgery, Pomeranian Medical Universityin Szczecin, Powstańców Wlkp. 72, 70-111 Szczecin, Poland; katarzyna.barczak@pum.edu.pl

**Keywords:** multiple myeloma (MM), chemokine, bone marrow, myeloma bone disease, cytokine

## Abstract

Multiple myeloma (MM) is a plasma cell neoplasm. Studies of chemokines in MM pathogenesis have primarily focused on CCR1 ligands such as CCL3 (MIP-1α), CXCL12 and its receptor CXCR4, as well as CXCR2 and CXCR3 ligands. However, the roles of the remaining 30 chemokines have been investigated much less frequently. This review compiles current knowledge on the significance of lesser-known chemokines in MM tumor processes, including CXCL13, CCR2 ligands (CCL2 [MCP-1], CCL7 [MCP-3]), CCL4, CCL5 (RANTES), CCL17, CCL20, CCL27, CCL28, and CX_3_CL1 (fractalkine). It describes their impact on bone destruction, bone marrow angiogenesis, chemoresistance, and the recruitment of cells into the MM niche, such as macrophages, myeloid-derived suppressor cells, and cytotoxic lymphocytes, along with their effects on mesenchymal stromal cells. A bioinformatic analysis highlights the significance of these chemokines in MM, and the possibility of targeting them in MM therapy is also considered.

## Simple Summary

This review compiles current knowledge on the significance of lesser-known chemokines in multiple myeloma (MM) tumor processes, including CXCL13, CCR2 ligands (CCL2 [MCP-1], CCL7 [MCP-3]), CCL4, CCL5 (RANTES), CCL17, CCL20, CCL27, CCL28, and CX_3_CL1 (fractalkine). It describes their impact on bone destruction, bone marrow angiogenesis, chemoresistance, and the recruitment of cells into the MM niche, such as macrophages, myeloid-derived suppressor cells, and cytotoxic lymphocytes, along with their effects on mesenchymal stromal cells.

## 1. Introduction

Multiple myeloma (MM) is a plasma cell malignancy. Most often, this cancer is located in the bones. However, in a small percentage of patients, MM cells also infiltrate soft tissues [1,2,3]. This is known as extramedullary disease. Each year, around 140,000 new cases are diagnosed globally, and approximately 100,000 patients die from the disease [4]. Two precursor states lead to MM: monoclonal gammopathy of undetermined significance (MGUS) [5,6] and smoldering MM (sMM) [7]. MM is marked by organ damage as defined by hypercalcemia, renal failure, anemia, and bone lesions (CRAB criteria) [8]. Although multiple lines of therapy exist, MM remains incurable and commonly relapses [1]. Research therefore focuses on understanding MM tumor mechanisms, clarifying why current therapies are insufficient, and identifying new therapeutic targets, with much attention on the MM bone marrow microenvironment.

Chemokines are one of the key components of this microenvironment. They are chemotactic cytokines [9], primarily responsible for guiding immune cells to sites of interest. Over time, additional roles have emerged, including modulation of immune responses [10] and communication between non-immune cells, for example, in muscle tissue [11]. Chemokines also affect hematopoietic stem cells (HSC), with the CXC motif chemokine ligand (CXCL)12–CXC motif chemokine receptor (CXCR)4 axis being especially well studied [12]. In solid tumors, chemokines contribute to immune cell recruitment [13,14], enhance the migratory capacity of tumor cells [14], and can influence cancer cell proliferation [15,16].

In humans, the chemokine system is made up of 43 cytokines, 18 receptors, and 4 atypical receptors [9]. These molecules are categorized into four subfamilies based on a conserved N-terminal motif: α-chemokines (CXC), β-chemokines (CC), γ-chemokines (XC), and δ-chemokines (CX_3_C). Most chemokine receptors are activated only by chemokines from the same subfamily, reflected in receptor nomenclature.

Chemokines are crucial to MM progression. Current research largely focuses on certain well-studied examples, including the CC motif chemokine receptor (CCR)1 ligands (particularly CC motif chemokine ligand (CCL)3, also called MIP-1α), CXCL12 (SDF-1) and its receptor CXCR4, CXCR2 ligands such as IL-8/CXCL8, and CXCR3 ligands (e.g., CXCL9/MIG and CXCL10/IP-10). However, the remaining 30 chemokines have been investigated far less in MM. The PubMed portal (as of 7 February 2026) contains only a few experimental articles on each of these 30 chemokines, or more precisely, chemokine axes. Of this group, CCL2 has the most articles, with 68 experimental articles. There are 87 experimental articles on CCL3, 103 articles on CXCL8, and 162 articles on CXCL12. This review therefore compiles existing data on these lesser-known chemokines in MM. The aim is to increase the scientific community’s interest in these chemokines. A bioinformatic analysis was also conducted using the KM-plotter portal (https://kmplot.com/analysis, accessed 1 September 2024) with the GSE4204 dataset and GSE24080 [16,17,18,19,20,21] to demonstrate their relevance, and the potential for targeting these chemokines in MM therapy is discussed.

Bioinformatic analysis compares the survival of MM patients with chemokine expression or chemokine receptor expression on MM cells. It should be noted that the bioinformatic analysis used has many disadvantages but also some advantages. The advantages include the ability to analyze almost any gene and to preliminarily indicate a possible link between the analyzed gene and MM cancer processes. The main disadvantage is that the raw data was obtained only from CD138+ MM cells. Unfortunately, the analysis does not provide information on the expression of chemokines and chemokine receptors on other bone marrow cells in MM patients. For this reason, in the case of chemokine expression alone, it is only possible to infer MM interactions with other MM bone marrow microenvironment cells through chemokine production. It is also possible to conclude from the chemokine receptor expression data whether a given chemokine acts on MM cells. However, it is not possible to conclude the interaction between non-MM cells in the MM bone marrow microenvironment.

## 2. α-Chemokines

α-chemokines contain the CXC motif at the N-terminus [9]. The roles of CXCL1–12 and their receptors (CXCR1–4) in MM have been well documented [22,23,24,25,26,27,28,29,30]. By contrast, few studies have examined CXCL13, CXCL14, CXCL16, and CXCL17 in MM.

### 2.1. CXCR5 and CXCL13

#### 2.1.1. The CXCL13–CXCR5 Axis

CXCL13 (previously BCA-1) binds the receptor CXCR5 [9,31]. CXCR5 is expressed on B cells and supports B-cell function in lymphoid follicles [32,33]. In solid tumors, CXCL13 can promote cancer cell migration and proliferation, recruit myeloid-derived suppressor cells and regulatory B cells, and also boost infiltration of CD4^+^ T cells, CD8^+^ T cells, and B cells, revealing both pro- and antitumor effects [33]. This axis similarly appears active in MM.

#### 2.1.2. The CXCL13–CXCR5 Axis in MM

MM cells express CXCR5 [34], although at lower levels than normal bone marrow B cells [35]. Compared to other chemokine receptors such as CXCR4, CXCR6, and CCR10, CXCR5 expression is relatively modest in MM [36]. Some studies have reported no CXCR5 expression on MM cells [37], or have observed it in only a fraction of patients [38]. In some cases, TP53 loss-of-function mutations can increase CXCR5 levels via elevated miR-19a [39]. Thus, CXCL13 may not normally act on MM cells unless certain mutations are present.

Despite this, MM patients have higher CXCL13 concentrations in bone marrow plasma and peripheral blood than healthy individuals [40], suggesting that CXCL13 may exert effects on non-MM cells within the marrow. Myeloid cells are a major source of CXCL13 [40], and interactions between MM cells and macrophages can increase CXCL13 expression in both. In MM cells, this upregulation is driven by transforming growth factor β (TGF-β), while in macrophages it can be reduced by inhibitors of Bruton’s tyrosine kinase, which may be exploited therapeutically. MM cells and mesenchymal stromal cells (MSCs) can also produce CXCL13 [34,40], and MSCs from MM patients display higher CXCL13 expression compared to healthy donors [34].

When CXCR5 is present on MM cells, CXCL13 can directly increase their proliferation [34] and migration [34,39]. Decreasing CXCL13 expression in mice lowers the MM burden [40], indicating a protumor role. Beyond its direct effects on MM cells, CXCL13 also elevates M2 macrophage levels in the bone marrow [35], providing immune protection to MM cells. Furthermore, CXCL13 contributes to bone destruction by enhancing osteoclast formation, partially through increasing receptor activator of NF-kappaB ligand (RANKL) expression in MSC and macrophages [40].

#### 2.1.3. The CXCL13-CXCR5 Axis as a Potential Therapeutic Target in MM

The CXCL13-CXCR5 axis may have therapeutic relevance in MM. CXCL13 induces chemoresistance to bortezomib by increasing B-cell leukemia/lymphoma-2 (Bcl-2) and P-glycoprotein (p-gp)/multiple drug resistance protein (MDR-1) in MM cells [34]. Bcl-2 is an anti-apoptotic protein that reduces bortezomib-induced cell death, while ATP binding cassette subfamily B member 1 (ABCB1)/P-glycoprotein (P-gp)/multi-drug resistance protein 1 (MDR-1) is a membrane transporter that exports bortezomib, lowering the cell’s sensitivity to this drug.

A bioinformatic analysis on the KM-plotter portal (https://kmplot.com/analysis accessed 1 September 2024) using the GSE4204 and GSE24080 dataset showed that, across all MM patients, higher CXCL13 or CXCR5 expression in MM cells does not correlate with overall survival [17,18,19,20,21]. In the HY molecular subgroup, however, higher CXCR5 expression is linked to poorer outcomes, and there is a trend (*p* = 0.055) toward worse outcomes for higher CXCR5 expression in the MF subgroup. In the MS subgroup, higher CXCL13 expression correlates with poorer survival. These findings suggest that, in HY patients, CXCR5-mediated interactions with the bone marrow microenvironment are most relevant, whereas in MS patients, CXCL13 production by MM cells and its effects on non-MM cells drive disease progression. Consequently, disrupting the CXCL13-CXCR5 axis with CXCR5 inhibitors or CXCL13-neutralizing antibodies may have therapeutic potential in these subgroups. It should be noted that this analysis does not account for interactions among non-MM cells, so it simplifies the broader cell–cell interactions that occur in MM.

In contrast, in the CD1 subgroup, higher CXCL13 expression in MM cells is associated with better survival, echoing certain antitumor effects observed for this chemokine in solid tumors [33].

### 2.2. CXCL14

CXCL14 was previously called BRAK [31]. Its receptor is not clearly defined, but potential targets include G-protein coupled receptor (GPR)85 [41], atypical chemokine receptor 2 (ACKR2) [42], insulin-like growth factor 1 receptor (IGF-1R) [43], low-density lipoprotein receptor-related protein 1 (LRP1) [44], and possibly CXCR4 [45], though the CXCL14–CXCR4 interaction is debated [46]. CXCL14 can bind cytosine-phosphate-guanine (CpG) DNA and serve as a carrier molecule [47], influencing dendritic cell function. Studies in solid tumors suggest it can either promote or inhibit tumor growth, depending on the tumor type and experimental model [48]. It can increase or reduce cancer cell proliferation, migration, and invasion and may facilitate cytotoxic lymphocyte infiltration [49]. It also exhibits angiostatic activity [50]. In chronic myeloid leukemia (CML), CXCL14 reduces the maintenance of leukemia-initiating stem cells, inhibiting disease progression [51].

Although its role in MM has not been investigated, the KM-plotter analysis of the GSE4204 and GSE24080 datasets revealed no correlation between CXCL14 expression in MM cells and survival for the overall patient group [17,18,19,20,21]. Within the HY subgroup, higher CXCL14 expression was linked to poorer outcomes, with a similar trend (*p* = 0.068) in the MF subgroup. In the PR subgroup, however, higher CXCL14 was associated with improved prognosis. These findings suggest that *CXCL14* can promote or suppress tumor growth, depending on the molecular subgroup. In the HY and MF subgroups, it appears to play a protumor role and may therefore be a potential therapeutic target.

### 2.3. CXCR6 and CXCL16

#### 2.3.1. The CXCL16-CXCR6 Axis

CXCL16 is synthesized in a membrane-bound form, which can then be cleaved to generate a soluble form. The membrane-bound form functions as an adhesion molecule for cells expressing CXCR6 [52]. CXCR6 is found on activated CD4^+^ T cells, activated CD8^+^ T cells, natural killer T (NKT) cells, and γδ T cells [9,53]. In solid tumors, the CXCL16-CXCR6 axis can either promote or inhibit tumor growth, depending on the specific tumor and model system [54]. The soluble form drives cancer cell migration, whereas the membrane-bound form limits migration by binding tumor cells [55].

#### 2.3.2. The CXCL16-CXCR6 Axis in MM

The importance of this axis in MM remains unclear. MM cells express CXCR6 [36], enabling them to bind and respond to CXCL16. Mesenchymal stromal cells also produce CXCL16 [36], potentially allowing MM cells to adhere to them via CXCL16-CXCR6. In addition, CXCL16 promotes MM cell adhesion to fibronectin [36].

**Figure 1:**
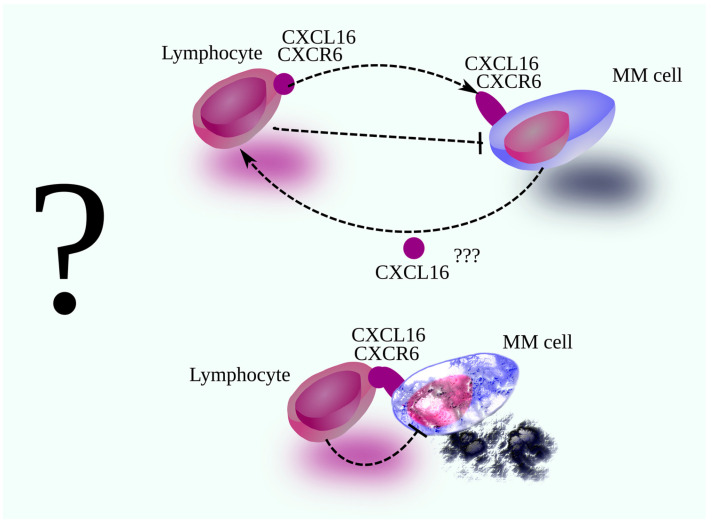
Anti-MM properties of the CXCL16-CXCR6 axis. Higher expression of CXCL16 and CXCR6 in MM cells is associated with a better prognosis in patients with MM. There are no precise studies explaining this relationship. Considering the properties of this axis, it can be theorized that this axis participates in the interaction of cytotoxic lymphocytes with MM cells. CXCL16 is a chemokine that causes lymphocyte chemotaxis. Therefore, it may induce lymphocyte migration to the vicinity of MM cells. The membrane form of CXCL16 and CXCR6 can also bind cells. This may lead to adhesion of MM cells with cytotoxic lymphocytes, facilitating the destruction of MM cells.

Although its precise function in MM is unknown, the KM-plotter analysis of the GSE4204 and GSE24080 dataset points to an antitumor role [17,18,19,20,21]. Higher CXCR6 or CXCL16 expression in MM cells correlates with better outcomes, most notably in the CD1 molecular subgroup for both molecules, in the PR subgroup for CXCR6, and in the CD2 subgroup for CXCL16. The CXCL16-CXCR6 axis is important in lymphocyte adhesion [9,53]. Therefore, it can be assumed that the antitumor properties of this axis in MM may be due to the interaction of MM cells with immune cells. These assumptions should be tested, particularly with regard to their use in CAR T cell therapy (Figure 1).

### 2.4. CXCL17

#### 2.4.1. CXCL17 and Its Receptor

Previously known as VCC-1, CXCL17 primarily signals through GPR35 [56], a receptor sometimes proposed for renaming as CXCR8. GPR35 can also be activated by other factors such as the serotonin metabolite 5-hydroxyindoleacetic acid (5-HIAA) [57], and there is evidence that GPR35 may not be the sole receptor for CXCL17 [58]. GPR35 is expressed on macrophages, monocytes, neutrophils, eosinophils, basophils, and dendritic cells [9], so CXCL17 can act on these cell types. It also binds glycosaminoglycans, potentially influencing other chemokines [59]. In solid tumors, CXCL17 may either inhibit or promote tumor progression, depending on the tumor context [58,60,61,62]. Its role in MM has not been explored experimentally.

#### 2.4.2. CXCL17 and Its Receptor in MM

A bioinformatic analysis on the KM-plotter portal (https://kmplot.com/analysis, accessed 1 September 2024) using the GSE4204 dataset found that CXCL17 likely has a protumor effect in MM [17,21]. An analysis demonstrated the unproven association of CXCL17 efficiency in MM cells with prognosis using the GSE24080 dataset [18,19,20,21]. However, differential results were obtained for the molecular subgroup of MM. Higher CXCL17 expression in MM cells was associated with worse outcomes overall and in the HY, LB, and MY molecular subgroups. This suggests that CXCL17 may be a promising therapeutic target in these subgroups (Table 1 and Table 2). In contrast, in the CD1 subgroup, higher CXCL17 expression in MM cells correlated with better prognosis.

Regarding its receptor, GPR35, expression in MM cells does not significantly affect survival in the overall patient group. However, in the PR subgroup, higher GPR35 expression is linked to better outcomes, while in the HY subgroup there is a borderline trend (*p* = 0.052) toward worse survival at higher expression. This pattern suggests a possible autocrine loop in which MM cells produce CXCL17 that then acts back on the same cells, enhancing tumor growth. In the LB and MY subgroups, such an autocrine relationship is less likely, and CXCL17 may act mainly on other cells in the MM microenvironment to promote tumor progression.

No direct experimental work has clarified the role of CXCL17 in MM, but its elevated expression in MM cells is tied to poorer outcomes, indicating it may be important for tumor progression. Further studies are needed to determine its function in the MM bone marrow microenvironment.

## 3. β-Chemokines

β-chemokines have a CC motif at the N-terminus [8]. In humans, they include CCL1 through CCL28, excluding CCL6, CCL9, CCL10, and CCL12, which are found in mice. These chemokines activate ten receptors: CCR1–CCR10. In MM, CCL3 (MIP-1α) and its receptor CCR1 have been most thoroughly investigated. Through their involvement in MM bone disease [63,64,65], CCL3 increases RANKL expression on osteocytes, which leads to bone loss [66]. Also, CCL3 and CCR1 receptor activation cause chemoresistance to various drugs in MM therapy, including dexamethasone [67], bortezomib [68,69] and melphalan [69]. CCL3 also impairs the function of megakaryocyte-erythroid progenitors in MM patients, contributing to anemia [70,71]. Other β-chemokines in MM have received less attention.

### 3.1. CCR2

#### 3.1.1. CCR2 and Its Ligands: CCL2, CCL7, CCL8, and CCL13

CCR2 ligands include CCL2 (MCP-1), CCL7 (MCP-3), CCL8 (MCP-2), and CCL13 (MCP-4) [9,31]. These factors act as chemoattractants for monocytes and macrophages [72,73,74]. CCL7 also binds CCR1, CCR3, and CCR5, and CCL13 can bind CCR1 and CCR3 [9], expanding their impact to a broader range of immune cells such as basophils and eosinophils [75]. In solid tumors, CCL2 promotes macrophage [76] and monocytic-myeloid-derived suppressor cell (M-MDSC) [77] infiltration. In acute myeloid leukemia (AML), CCL2 can contribute to thymic atrophy and immunodeficiency [78].

#### 3.1.2. CCR2 and Its Ligands: CCL2, CCL7, CCL8, and CCL13 in MM

Although the importance of CCR2 ligands has been recognized, it is less thoroughly characterized than the roles of CXCL12, CXCL8/IL-8, or CCL3. MM cells express CCR2 [79,80,81], though the fraction of patients with CCR2-positive MM cells varies from about one-quarter [38] to more than half [23]. In peripheral blood, more than half of MM cells are CCR2-positive [38], suggesting a mechanism for their release from bone marrow. In patients with active disease, fewer exhibit CCR2 expression [23]; lack of CCR2 correlates with higher β-2-microglobulin (B2M) and C-reactive protein (CRP) levels and lower hemoglobin [23]. Even in cases where MM cells express CCR2, typically only about one-quarter of these cells show it [82].

Serum levels of CCL2 do not uniformly differ between MM patients and healthy controls [26,83]; some studies report lower levels [75,76], while others find higher levels [84,85,86,87,88]. Nevertheless, CCL2 levels positively correlate with bone lesions, renal impairment [83], and anemia [26,83], as well as total vascular area in the bone marrow [83], linking CCL2 to bone marrow angiogenesis in MM. Elevated CCL2 in serum is also associated with poorer prognosis [89]. Another CCR2 ligand, CCL13, is elevated in MM patients [90], correlates positively with B2M, and shows an inverse relationship with white blood count (WBC) [90]. These findings point to a strong link between disease severity and CCR2 ligands.

Levels of CCL2 in the bone marrow plasma of MM patients are higher than in healthy individuals [87,91,92] and MGUS patients [92]. Bone marrow cells in MM also produce more CCL2 than those in MGUS and healthy controls [93]. Macrophages appear to be the primary source of CCL2 in the MM bone marrow [88], and MM cells boost this chemokine’s expression in macrophages. Bone marrow macrophages further increase CCL2 production through erythropoietin receptor (Epo-R) activation [94], and Epo-R expression is higher in MM than MGUS.

All three CCR2 ligands are also expressed by MSCs in the bone marrow [80]. MM cells can elevate at least CCL2 production in MSCs [92,95,96], partly via the transfer of miR-146a in MM cell–derived exosomes. One study did not detect such an effect [97]. Bone marrow endothelial cells [82] and osteoblasts [98] produce CCL2 under MM cell stimulation, and osteoclasts generate CCR2 ligands such as CCL2, CCL7, and CCL8 [99]. Osteoclasts have higher CCL7 and CCL8 expression than MM cells, suggesting they may be a more important source of these chemokines.

In some patients, MM cells themselves can also produce CCL2 [91], though MM cells are generally not the main source in bone marrow [92]. Interacting with M2-type macrophages in bone marrow increases CCL2 production in MM cells [100,101] and also raises CCR2 expression, intensifying the effect of this axis on MM cells [101]. Tumor necrosis factor-α (TNF-α) can similarly elevate CCL2 expression in MM cells [101].

#### 3.1.3. Association of CCR2 and Its Ligands with MM Prognosis

Studies relating the expression and levels of CCR2-axis components to MM prognosis have yielded mixed findings. A higher level of CCR2 on MM cells is linked to poorer survival [23], suggesting that receptor activation is important in MM pathogenesis. Conversely, elevated CCL2 expression in MM cells correlates with better outcomes [102,103], although MM cells are not the main source of CCL2 in the bone marrow [92].

A bioinformatic analysis on the KM-plotter portal (https://kmplot.com/analysis, accessed 1 September 2024) using the GSE4204 [17,21] and GSE24080 datasets [18,19,20,21] suggests that this axis may overall have an antitumor effect when considering all MM patients. According to the GSE4204 dataset, higher expression of CCR2, CCL2, and CCL8 in MM cells is associated with better survival. However, these correlations depend on the molecular subgroup. In the LB subgroup, higher CCR2 expression predicts poorer outcomes. Similar patterns emerge for CCR2 ligands: higher CCL7 expression is linked to worse survival in the PR and MY subgroups, CCL8 in the MS subgroup, and CCL13 in the MF subgroup. There is also a trend (*p* = 0.06) toward worse survival at higher CCL13 expression in the LB and MS subgroups. Thus, in four of the eight subgroups associated with adverse outcomes, higher expression of a specific CCR2 ligand in MM cells correlates with poorer survival. This supports the idea that CCR2 ligands secreted by MM cells may mainly affect other cell types rather than MM cells themselves.

#### 3.1.4. Role of CCR2 Ligands in MM Tumor Processes

CCR2 ligands appear to influence MM development and progression. Higher circulating CCL8 in healthy individuals is associated with an increased risk of MM [104], while lower circulating CCL7 corresponds to a higher MM risk [105,106]. CCL7 has a broad range of immune activities [107], strongly affecting basophils and eosinophils [75], and can also act on CCR5-expressing T cells and natural killer (NK) cells [9]. These observations may point toward a weakened immune response as a factor in MM initiation, and they also suggest that CCL8 may contribute to MM onset and growth.

CCR2 ligands can act on MM cells or on tumor-associated cells in the bone marrow. Depending on the cell line, they can either increase [99] or not affect [88] MM cell proliferation. Cells producing CCR2 ligands attract MM cells, driving their migration toward MSCs [80] and osteoclasts [99]. Though this process depends on other factors, such as CCL3, MM cell migration to these cells promotes adhesion between MM cells and MSCs or osteoclasts and heightens interactions between the two cell types. CCR2 ligands can also promote MM cell egress from the bone marrow [38], explaining why peripheral blood contains a higher proportion of CCR2-expressing MM cells than the bone marrow.

The CCR2 axis also appears to act on tumor-associated cells in the bone marrow. CCR2 ligands, particularly CCL2, recruit macrophages [92], which then polarize toward an immunosuppressive M2 phenotype—a process partly driven by CCL2 [88]. These M2 macrophages protect MM cells from immune surveillance and immunotherapies.

Finally, CCR2 ligands may contribute to bone destruction in MM. Elevated serum CCL2 correlates with bone lesions in some studies [83], though not all [89]. CCL2 can induce osteoclastogenesis by counteracting MM cell-derived interleukin-10 (IL-10), which would otherwise reduce RANKL expression on monocytes [95]. This effect is especially pronounced when MSCs are present, as MM cells increase CCL2 expression in MSCs [95]. CCL2 itself also elevates RANKL in monocytes [95], indicating that CCR2 ligands are among the factors driving myeloma bone disease.

#### 3.1.5. CCR2 Ligands and MM Therapy

CCR2 ligands may have therapeutic relevance in MM. CCL2 contributes to treatment side effects, such as peripheral neuropathy, which occurs in about half of MM patients treated with agents like bortezomib [108]. Individuals who have higher post-chemotherapy levels of CCL2 and proinflammatory cytokines such as interferon-γ (IFN-γ) and interleukin-1β (IL-1β) are more likely to develop peripheral neuropathy [109]. This effect may involve inflammatory responses in the dorsal root ganglion [110], where CCL2 expression is elevated [111]. Increased expression of activating transcription factor 3 (ATF3) and activation of c-Jun upregulate the CCL2 gene promoter in dorsal root ganglia, causing mechanical allodynia in rat models [111]. These findings suggest that CCR2 inhibitors could help reduce certain chemotherapy-related side effects in MM.

CCR2 ligands can also promote chemoresistance. CCL2 itself does not directly affect the sensitivity of MM cells to agents like bortezomib [88], but it acts on macrophages, inducing an M2 phenotype that shields MM cells from bortezomib and melphalan. M2 macrophages are immunosuppressive, protecting MM cells from immune-based therapies.

Some CCR2 inhibitors have undergone in vivo testing for antitumor activity in MM. One example is CCX140-B [88]. This compound did not affect tumor growth or MM cell susceptibility to bortezomib. However, in the presence of macrophages within the MM microenvironment, the CCR2 inhibitor enhanced MM cell sensitivity to bortezomib [88]. Combining a CCR2 inhibitor with standard MM therapy such as bortezomib could thus provide therapeutic benefits.

### 3.2. CCR3

#### 3.2.1. CCR3 and Its Ligands: CCL11, CCL24, CCL26

CCR3 ligands include CCL11, CCL24, and CCL26, as well as CCL4, CCL5, CCL7, CCL13, CCL15, CCL23, and CCL28 [9]. CCR3 is primarily found on eosinophils [8]. Its ligands are therefore key eosinophil chemoattractants [112]. CCL11, CCL24, and CCL26 were originally named eotaxin, eotaxin-2, and eotaxin-3, respectively [31]. By driving eosinophil infiltration, this axis is involved in allergic conditions [113]. In solid tumors, CCR3 activation can increase proliferation and migration of cancer cells [114] and promote angiogenesis by directly affecting endothelial cells [115]. Conversely, infiltrating eosinophils can curb angiogenesis and tumor growth [116].

#### 3.2.2. CCR3 and Its Ligands in MM

CCR3’s significance in MM remains poorly understood. MM cells express CCR3 [36] but at lower levels than other chemokine receptors such as CXCR4, CXCR6, and CCR10 [36]. Another study reported CCR3 expression in only 1 out of 10 MM patients [38]. Serum CCL11 levels do not differ between MM patients and healthy individuals [26,86], and bone marrow plasma levels of CCL11 are similarly unchanged in MM [91].

A bioinformatic analysis on the KM-plotter portal (https://kmplot.com/analysis accessed 1 September 2024) using the GSE4204 dataset suggests that bone marrow microenvironmental signals acting through CCR3 on MM cells are important [17,21]. Higher CCR3 expression in MM cells correlates with poorer outcomes, particularly in the HY and LB subgroups, with a borderline trend (*p* = 0.058) in the MF subgroup. However, higher CCL11 and CCL24 expression in MM cells is linked to better survival, implying a protective role when these chemokines are secreted by MM cells. This pattern does not hold in all molecular subgroups. For instance, in the HY subgroup, elevated CCL11 expression correlates with worse outcomes, and in the MF and MY subgroups, higher CCL26 expression is also associated with worse outcomes. In the HY subgroup, MM cells may secrete CCL11 that acts autocrinely, potentially presenting a therapeutic target, while in the MF subgroup, CCL26 might play a similar role. These findings suggest that CCR3 inhibitors may be beneficial against MM, but perhaps only for HY and MF subgroups.

The poorer prognosis in patients whose MM cells express more CCR3 indicates that this receptor may be important in MM tumor mechanisms, but experimental data are lacking. Further research on how CCR3 functions in MM cells is necessary to clarify its role in the bone marrow microenvironment.

### 3.3. CCR4

#### 3.3.1. CCR4 and Its Ligands: CCL17 and CCL22

CCR4 ligands are CCL17 and CCL22 [9]. CCR4 is expressed on CD4^+^ T cells, CD8^+^ T cells, NKT cells, regulatory T cells (T_reg_), and type 2 helper T (Th2) cells [9,117]. Because Th2 cells express CCR4, this axis contributes to allergic responses. In solid tumors, CCR4-mediated signaling can recruit T_reg_ into the tumor niche [118,119,120]. However, it may also facilitate infiltration of cytotoxic lymphocytes into the tumor, producing an antitumor effect [121].

#### 3.3.2. CCR4 and Its Ligands in MM

The CCL17/CCL22-CCR4 axis appears to exert an antimyeloma effect. CCR4 ligands contribute to immune responses, so MM cells with high expression of these ligands are more likely to be targeted by the host immune system—an effect particularly relevant in early-stage MM [122]. Later, as the MM bone marrow microenvironment matures, T_reg_ cells proliferate [123] and inhibit immune responses against MM. Enhancing immune activity and reducing T_reg_ function may thus be a viable therapeutic strategy, supported by mouse model studies [122].

Patients with MM have higher CCL17 concentrations in their blood compared to healthy individuals [124]. CCL17 may be involved in MM pathogenesis by inhibiting osteoblast differentiation [124], which could worsen bone destruction in MM. Elevated blood CCL17 in MM patients also predicts response to lenalidomide therapy [125], though it can indicate allergic reactions to this agent (manifested as skin rashes) [125]. Lenalidomide-based chemotherapy raises serum CCL17, which recruits Th2 cells [117] responsible for allergic-type responses, whereas bortezomib-based therapy decreases serum CCL17 [125].

A bioinformatic analysis on the KM-plotter portal (https://kmplot.com/analysis accessed 1 September 2024) using the GSE4204 dataset shows that the CCL17/CCL22-CCR4 axis plays an antitumor role in MM [17,21]. Higher CCL17 expression in MM cells correlates with better outcomes, suggesting that CCL17 may not recruit T_reg_ but instead attract cytotoxic lymphocytes into the bone marrow tumor. Analyses using the GSE24080 dataset showed trends (*p* < 0.10) of better prognosis with higher expression of this chemokine in MM cells [18,19,20,21]. According to the GSE4204 dataset, higher CCR4 expression in MM cells trends (*p* = 0.077) toward worse prognosis, hinting that CCR4 activation on MM cells could contribute to tumor processes.

Because CCL17 in MM is linked to improved outcomes, a potential therapeutic approach would be to increase its levels in the MM bone marrow microenvironment. One example might be chimeric antigen receptor-modified (CAR) T cells engineered to overexpress CCL17, which could elevate local CCL17 concentration in the bone marrow, promote cytotoxic lymphocyte infiltration, and enhance the immune response against MM.

### 3.4. CCR5

#### 3.4.1. CCR5 and Its Ligands CCL4 and CCL5

CCR5 can be activated by multiple chemokines, including CCL3, CCL4, CCL5, CCL7, CCL11, CCL14, and CCL16 [9]. In this section, CCL4 and CCL5 are emphasized. These chemokines also activate CCR1 and CCR3. CCR5 is expressed on B cells, CD4^+^ T cells, CD8^+^ T cells, NKT cells, NK cells, dendritic cells, monocytes, and macrophages [8]. It supports immune system functions by promoting the infiltration of cytotoxic lymphocytes [126,127]. In solid tumors, CCL4 and CCL5 drive tumor infiltration by NK cells, CD4^+^ T cells, and CD8^+^ T cells, producing antitumor effects [128,129,130]. However, these chemokines can also recruit T_reg_ [130], expand myeloid-derived suppressor cells (MDSC) in the bone marrow [131], and promote angiogenesis [132].

#### 3.4.2. CCR5 and CCL5 in MM

Genetic polymorphism studies suggest that the CCL5-CCR5 axis may not initiate MM but can influence disease progression during therapy. In a Polish cohort, certain CCL5 genotypes (rs2280789, rs2280788, rs2107538) do not correlate with MM incidence, but CG+CC genotypes at rs2280788 and CC at rs2107538 are associated with a higher risk of relapse [133]. Conversely, patients with the major genotypes show better outcomes with thalidomide or bortezomib and have higher serum CCL5, indicating a favorable response to chemotherapy [133].

Studies also show CCR5 expression on MM cell lines [134] and in patient-derived MM cells [135], though only about one-third of patients have CCR5-positive MM cells [38]. Expression levels are lower than those of some other receptors like CCR1 [135], yet they can be enhanced through interactions with tumor-associated macrophages [101].

Some reports indicate higher serum CCL5 in MM [26], while others find no difference compared to healthy controls [86]. No significant changes in bone marrow plasma CCL5 have been identified among MM, MGUS, and healthy groups [92], though further confirmation is needed. MM cells themselves can secrete CCL5 [136], and certain cell lines produce it [91,137]. Within the bone marrow, interactions with macrophages boost CCL5 production by MM cells [100]. CCL5 is also higher in differentiated MM cells than in side population (SP) MM cells [138]. MSCs represent another potential source of CCL5; MM cells can induce MSCs to produce it by transferring miR-146a through exosomes [96].

Elevated CCL5 in the bone marrow plasma of MM patients with extramedullary disease indicates that it may promote MM cell egress from the marrow [139]. CCR5 also contributes to bone marrow angiogenesis driven by MM; inhibiting CCR5 reduces angiogenesis, although the key ligand remains unclear [140]. CCL5 can recruit M-MDSCs and enhance their immunosuppressive activity, especially when combined with exosomes carrying miR-106a-5p from MM cells [141,142]. Immunomodulatory drugs such as lenalidomide and pomalidomide lower CCL5 expression in MM cells, reducing MDSC recruitment and immunosuppression [141].

#### 3.4.3. CCL4 in MM

Serum CCL4 levels are higher in MM patients than in healthy individuals [26,86]. Bone marrow plasma CCL4 levels are elevated in MM and MGUS compared to healthy controls [87], although one study did not replicate this finding [92]. MM cells produce CCL4 [136,143], and adhesion to the extracellular matrix (ECM) particularly via integrin β7 further increases its expression [144].

Greater CCL4 production by MM cells correlates with more severe bone destruction [143,145], suggesting a role in myeloma-related bone lesions. Osteoclast precursors respond to CCL4 via CCR5, which boosts RANK expression and osteoclast activity [143]. Anticancer drugs can alter CCL4 expression; for example, bortezomib reduces its levels in MM cells [146]. Lower serum CCL4 predicts a better response to bortezomib [26], and a stronger CCL4 response in peripheral blood after lipopolysaccharide (LPS) and zymosan A stimulation suggests a favorable outcome to bortezomib [147]. Since CCL4 supports NK cell function [148] and bortezomib enhances immune-mediated tumor suppression [149], a robust CCL4 response may reflect the immune system’s ability to control MM when exposed to bortezomib.

#### 3.4.4. Bioinformatic Analysis of CCR5, CCL4, and CCL5 in MM

A KM-plotter portal analysis (https://kmplot.com/analysis, accessed 1 September 2024) using the GSE4204 [17,21] and GSE24080 datasets [18,19,20,21] shows that the described axis may not be a convenient therapeutic target. CCL4 and CCL5 expression in MM cells does not correlate with prognosis for all patients, although higher CCL5 in the LB subgroup is associated with improved survival and in the MF subgroup with worse survival. CCR5 expression in MM cells is not linked to outcomes overall, but in CD2, HY, and LB subgroups, higher CCR5 correlates with poorer survival. These data suggest that in certain molecular subgroups, CCR5 activation on MM cells plays a key role in tumor progression, making CCR5 a potential therapeutic target in these settings.

### 3.5. CCR6 and Its Ligand CCL20

CCR6 is activated by the ligand CCL20 [9] and is expressed on B cells, T_reg_, basophils, and dendritic cells. Through its action on T helper type 17 (Th17) cells and T_reg_, the CCL20–CCR6 axis helps maintain immune homeostasis [150]. In solid tumors, CCL20–CCR6 promotes the recruitment of T_reg_ [151,152] and Th17 cells [153] to the tumor niche.

In MM, CCR6 is expressed at lower levels on MM cells than on normal bone marrow B cells [35]. Compared to CXCR4, CXCR6, and CCR10, its expression is much weaker [36], and some studies detect CCR6 only in a subset of patients [38] or certain MM cell lines [154].

By contrast, CCL20 is found on the MM cells of only about 20% of patients [155], and its presence often correlates with CCR6 expression, hinting at a possible autocrine loop. Bone marrow plasma levels of CCL20 are higher in MM than in MGUS [87,155,156], sMM [156], or healthy controls [87]. Patients with MM-related osteolysis have higher CCL20 concentrations than those without bone destruction [155,156], suggesting that CCL20–CCR6 may contribute to osteolytic lesions in MM. MM cells upregulate CCL20 and CCR6 in bone marrow osteoprogenitor cells, osteoblasts, and osteoclasts [155]. In osteoprogenitor cells and osteoblasts, proinflammatory cytokines IL-1β and TNF-α (but not interleukin 6 (IL-6)) further boost CCL20–CCR6, increasing RANKL expression and enhancing osteoclastogenesis, leading to bone destruction [155].

This axis may also affect drug sensitivity. In MM lines resistant to elotuzumab or lenalidomide, CCL20 levels are lower compared to sensitive lines; similarly, patients whose MM is resistant to these drugs have reduced circulating CCL20 [157]. Because CCL20 acts on T cells, B cells, and dendritic cells (DC) [158], reduced CCL20 might compromise immune responses during treatment. In vitro data suggest a direct effect on MM cells as well [157], although the mechanism remains unclear.

A bioinformatic analysis (KM-plotter portal, https://kmplot.com/analysis, accessed 1 September 2024) using the GSE4204 [17,21] and GSE24080 datasets [18,19,20,21] indicates no strong overall association between CCL20–CCR6 expression in MM cells and survival. However, in the HY molecular subgroup, higher CCR6 levels correlate with poorer outcomes, implying that CCR6 activation may be particularly relevant to tumor progression in this subgroup.

### 3.6. CCR7 and Its Ligands CCL19, CCL21

CCR7 ligands include CCL19 and CCL21 [9]. CCR7 is primarily expressed by T cells but also appears on dendritic cells and B cells [9,159]. Its key function is guiding lymphocytes to secondary lymphoid organs [160]. In solid tumors, CCL19/CCL21–CCR7 recruits CD4^+^ and CD8^+^ T cells into the tumor, conferring antitumor effects [161,162,163], yet it can also attract T_reg_ [164]. Elevated ligand levels in lymph nodes enable cancer cells that express CCR7 to metastasize there [165].

Roughly half of MM patients have CCR7-positive MM cells [166], though its expression is considerably lower than that of CXCR4, CXCR6, or CCR10 [36]. Other studies do not detect CCR7 on MM cells at all [37,38]. Little is known about how CCL19/CCL21–CCR7 influences MM, though more CCR7-positive MM cells are found in patients with extramedullary disease [166], suggesting a role in allowing MM cells to exit the bone marrow or spread beyond it.

CCR7 ligands facilitate T-cell activity [160,161,162,163] and may enhance adoptive cell therapy. BCMA-7 × 19 CAR T cells, for instance, target B cell maturation antigen (BCMA), an antigen on MM cells and chronic lymphocytic leukemia (CLL) B cells [167,168], and also overexpress interleukin-7 (IL-7) and CCL19 [169]. IL-7 increases CAR T-cell proliferation, while CCR7 ligands provide additional T-cell costimulation [170]. Compared to standard BCMA-targeted CAR T cells, these enhanced cells show greater expansion and cytotoxicity without raising interleukin-2 (IL-2), IFN-γ, or granulocyte-macrophage colony-stimulating factor (GM-CSF) levels, thus avoiding neurotoxicity or severe cytokine release syndrome. In clinical trials (NCT03778346), BCMA-7 × 19 CAR T cells have been highly effective against relapsed/refractory MM, causing only grade 1 cytokine release syndrome and some neutropenia [169].

**Figure 2:**
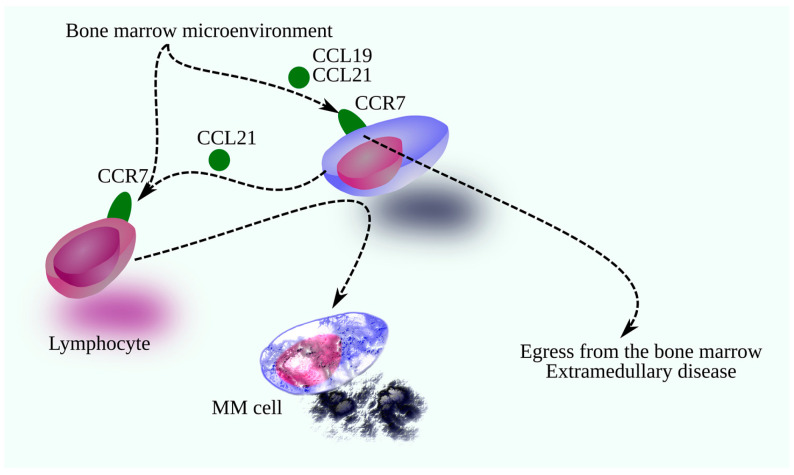
The importance of the CCL19/CCL21-CCR7 axis in MM. The CCR7 receptor on MM cells has protumor properties. However, CCL21 expression in MM cells has antitumor properties. The antitumor properties of CCL21 may be linked to cytotoxic lymphocytes that destroy MM cells. Activation of the receptor on MM cells may be associated with MM cell migration and expulsion from the bone marrow, leading to extramedullary disease and a poorer prognosis.

KM-plotter analysis (GSE4204 and GSE24080 datasets) [17,18,19,20,21], https://kmplot.com/analysis, accessed 1 September 2024) indicates that CCL19/CCL21–CCR7 has antitumor potential in MM overall, as higher CCL21 expression correlates with better survival [17,18,19,20,21]. However, in the MS molecular subgroup, higher CCL19 is linked to worse outcomes, although CCL21 is not prognostic. This discrepancy suggests the axis could be therapeutically targeted in MS patients. Meanwhile, higher CCR7 levels in MM cells from the LB and MY subgroups correlate with poorer survival, with a similar trend in MF and CD1, implying that CCR7 activation on MM cells may be protumor in these contexts. While CCL21 expression in MM cells has an antitumor effect (Figure 2), CCL21 is a chemokine that acts on lymphocytes [9,159,160]. Therefore, it can be assumed that this axis is involved in the interaction and control of MM cells by the immune system.

### 3.7. CCR8, CCL1, and CCL18

CCR8 is activated by CCL1 and CCL18 [9,171]. Because CCR8 is expressed on Th2 cells [172,173], these ligands are involved in allergic responses. CCR8 is also found on endothelial cells, and its activation promotes angiogenesis [174]. In adult T-cell leukemia (ATL), CCL1 provides an autocrine anti-apoptotic signal [175]. Moreover, CCR8 ligands can recruit T_reg_ to the tumor niche [176].

CCL18 is recognized as a marker of M2-polarized macrophages [177,178]. In solid tumors, tumor-associated macrophages (TAM) are the primary source of CCL18 [179,180,181]. Although it weakly activates CCR8 [182], CCL18 can also bind phosphatidylinositol transfer protein membrane-associated 3 (PITPNM3) [183], CCR6 [184], and GPR30 [185]. PITPNM3 appears to be the main receptor. Because PITPNM3 is expressed on endothelial cells, CCL18 can induce angiogenesis [186]. Via PITPNM3, it also attracts naïve CD4^+^ T cells to the tumor niche, where they can differentiate into T_reg_ [187].

#### CCR8, CCL1, and CCL18 in MM

Currently, no data are available on CCL1 or CCR8 in MM. Research on CCL18 in MM is limited. Serum levels of CCL18 are higher in MM patients than in MGUS patients or healthy controls [188], increasing with higher International Staging System (ISS) stages. This makes CCL18 a potential marker of disease progression. Even MGUS patients display higher CCL18 than healthy individuals. Serum CCL18 levels correlate with renal dysfunction and hypercalcemia, reflecting MM-related bone damage [188]. High serum CCL18 also indicates worse outcomes in MM [188].

In the bone marrow, osteoclasts are the main source of CCL18 [99], although MM cells produce smaller amounts. CCL18 promotes MM cell migration but does not affect proliferation [188]. While higher CCL18 levels correlate with unfavorable prognosis, the exact molecular mechanisms remain unclear.

A bioinformatic analysis (KM-plotter portal, https://kmplot.com/analysis; accessed 1 September 2024) using the GSE4204 [17,21] and GSE24080 datasets [18,19,20,21] found that higher CCL1 and CCL18 expression in MM cells, considered across all patients, is linked to better outcomes. In the LB molecular subgroup, however, elevated CCL18 correlates with worse survival, suggesting context-dependent effects. CCR8 expression in MM cells is not tied to prognosis overall, while PITPNM3 (the main CCL18 receptor) shows a borderline (GSE4204 dataset *p* = 0.058; GSE24080 dataset *p* = 0.088) association with improved survival. In the MF subgroup, higher PITPNM3 is linked to worse outcomes, implying that this receptor could be a therapeutic target there. It should be noted that, in solid tumors, TAMs (not cancer cells) are the main producers of CCL18 [179,180,181], and a similar pattern may occur in MM.

### 3.8. CCR9 and CCL25

CCR9 is activated by CCL25 [9], a chemokine abundantly expressed in the thymus and small intestine, where it regulates the migration of activated lymphocytes [189]. In solid tumors, CCL25 can promote infiltration by cytotoxic CCR9^+^CD8^+^ T cells [190], but it can also recruit MDSCs [191] and affect TAMs in the tumor niche [192].

#### CCR9 and CCL25 in MM

The role of the CCL25–CCR9 axis in MM is poorly understood. Some cell line studies indicate that MM cells do not express CCR9 [134], although they can secrete CCL25 [137]. In the bone marrow, MM-derived CCL25 attracts MSCs [137].

A KM-plotter analysis (GSE4204 and GSE24080) [18,19,20,21] datasets, https://kmplot.com/analysis; accessed 1 September 2024 [17,21] suggests that CCL25 produced by MM cells may not be of significant importance for MM. CCR9 expression in MM cells does not correlate with outcomes, yet higher CCL25 levels in the PR, LB, MF, and MY subgroups predict worse survival. There is also a trend (*p* = 0.074) toward poorer prognosis with higher CCL25 expression overall. This points to CCL25–CCR9 as a potential therapeutic target, although its exact role—possibly acting on TAMs, MDSCs, or supporting MM cell proliferation and migration—remains unclear.

### 3.9. CCR10, CCL27, and CCL28

CCR10 is activated by CCL27 and CCL28 [9], and CCL28 can also activate CCR3. These chemokines act on T helper type 22 (Th22) cells, IgA-secreting plasma cells, and small subsets of preactivated or memory-like T cells in circulation. CCL27 recruits lymphocytes to the skin, while CCL28 attracts them to mucosal tissues [193,194] and helps maintain the hematopoietic stem cell pool [195]. In solid tumors, CCR10 ligands can be antitumor by recruiting cytotoxic lymphocytes, including NK cells [196]. They may also exert protumor effects by recruiting T_reg_ [197]. Furthermore, CCL28 activates CCR3 on endothelial cells to promote angiogenesis [198] and CCR10 on lymphatic endothelial cells to stimulate lymphangiogenesis [199].

#### CCR10 and Its Ligands CCL27 and CCL28 in MM

Patients with MM have higher CCL27 levels in circulation than healthy individuals [90]. A similar pattern is observed in bone marrow plasma [200], though CCL27 levels there do not correlate with tumor burden. In contrast, CCL28 levels in the bone marrow are much lower, and about half of MM patients show no detectable CCL28 at all [200]. Within the bone marrow, CCL27 is produced mainly by MM cells [200], while MSCs express CCL28 [36]. Elevated CCL27 in bone marrow plasma is associated with worse outcomes, and MM cells also express CCR10 [36]. Higher CCR10 expression correlates with poorer prognosis [201] and increases in relapsed MM, suggesting a significant role for the CCL27-CCR10 axis in disease progression.

**Figure 3:**
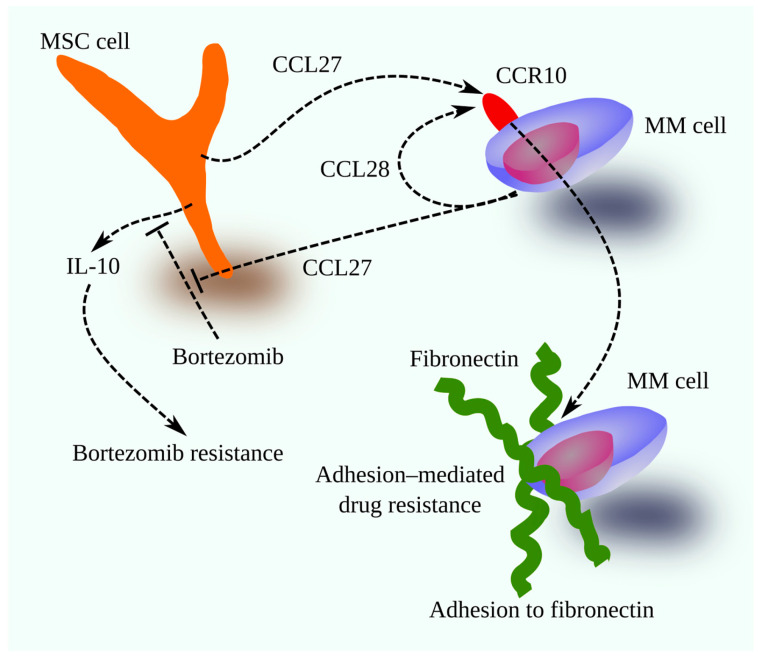
The CXCL27/CCL28-CCR10 axis in tumor mechanisms in MM. In the MM bone marrow microenvironment, the sources of CCR10 ligands are MSCs and MM cells. The former produce CCL27. MM cells, in turn, are a source of CCL28, although they can also produce CCL27. Activation of CCR10 on MM cells increases adhesion to fibronectin, leading to CAM-DR. Activation of CCR10 by CCL27 on MSCs also prevents bortezomib from affecting IL-10 expression in these cells. This leads to higher levels of IL-10, leading to bortezomib resistance.

CCL28 promotes MM cell adhesion to fibronectin [36], contributing to cell adhesion–mediated drug resistance (CAM-DR). The CCL27/CCL28-CCR10 axis also drives chemoresistance, particularly through CCL27-induced bortezomib resistance [200]. Bortezomib normally reduces IL-10 in the bone marrow, but CCL27 released by MM cells activates CCR10 on MSCs, preventing IL-10 downregulation. Moreover, bortezomib increases CCL27 expression in MM cells [200], heightening resistance in subsequent treatment cycles. Combining bortezomib with a CCR10 inhibitor could offer a new therapeutic strategy (Figure 3).

This axis is being explored as a potential therapeutic target. One approach involves CAR T cells engineered to target CCR10 [201]. These CAR T cells detect CCR10 using a CCR27-based recognition domain and carry a CCR10 gene knockout to avoid attacking each other. In vitro, they recognize and kill MM cells [201]. However, their clinical use may be limited because CCR10 is not exclusive to MM cells; it is also expressed on hematopoietic stem cells [195], raising concerns about collateral damage to healthy stem cells.

Bioinformatic analysis (KM-plotter portal, accessed 1 September 2024) of the GSE4204 [17,21] and GSE24080 datasets [18,19,20,21] suggests that while the CCR10 axis is associated with tumorigenic mechanisms in MM, higher CCL27 expression in MM cells is linked to better outcomes overall. However, analyses using the GSE24080 dataset did not confirm this. While elevated CCL28 expression in MM cells is associated with a poorer prognosis (Table 3, Table 4 and Table 5), the same is true for increased expression of the CCR10 receptor on MM cells. Specifically, LB subgroup patients with higher CCL28 experience worse survival, with a weaker trend (*p* = 0.063) in the HY subgroup. A similar pattern emerges for CCL27 in the HY and LB subgroups, indicating that in these subgroups, CCR10 ligands likely promote tumor progression within the bone marrow microenvironment. Higher CCR10 expression on MM cells is linked to poorer outcomes in the PR, HY (*p* = 0.072 trend), LB, MF, and MS subgroups but shows the opposite pattern in MY. Overall, these findings highlight CCR10 as a potential therapeutic target, particularly in the HY and LB subgroups of MM.

## 4. γ-Chemokines

### 4.1. XCR1 and Its Ligands XCL1 and XCL2

Ligands for XC motif chemokine receptor 1 (XCR1) are XC motif chemokine ligands (XCL)1 and XCL2 [9], and XCR1 is expressed only on dendritic cells [9,202]. Because of this, the XCL1–XCR1 axis is involved in immune responses. In solid tumors, increased XCL1 expression supports antitumor immunity [203], and higher XCR1 expression in tumors often correlates with a better prognosis in many solid malignancies [204].

### 4.2. XCR1 and Its Ligands XCL1, XCL2 in MM

Knowledge of the XCL1/XCL2–XCR1 axis in MM is limited. XCL1 appears to exert an anti-MM effect by promoting neutrophil infiltration in the bone marrow, as these cells express XCR1 [205]. The recruited neutrophils initiate an immune cascade that ultimately increases neutrophils, CD4^+^ T cells, and CD8^+^ T cells, producing anti-MM activity.

However, there is also evidence that this chemokine axis may have protumor properties in MM. A subset of type I conventional dendritic cells (cDC1s) described as CD11c^+^CD8a^+^XCR1^+^CD11b^−^ is recruited into the MM bone marrow microenvironment [206]. These cells can contribute to exhausted CD8^+^ T cells and an increase in T_reg_, though it is unclear whether XCL1/XCL2–XCR1 or another axis (for example, CCR7 or CXCR4) is responsible.

A bioinformatic analysis using the KM-plotter portal (https://kmplot.com/analysis, accessed 1 September 2024) with the GSE4204 dataset [17,21] suggests that XCR1 on MM cells plays a role in tumor processes. Higher XCR1 expression in MM cells is linked to worse outcomes, particularly in the CD2, PR, and HY molecular subgroups, while this relationship is reversed in CD1 and MY. In addition, elevated XCL1 expression in MM cells correlates with poorer outcomes in HY and, as a trend (*p* = 0.072), in PR and (*p* = 0.094) in LB. This suggests that MM cells might drive an autocrine XCL1–XCR1 loop. Hence, XCL1–XCR1 could be a therapeutic target in MM. Nevertheless, since it typically supports immune responses against pathogens and tumor cells, inhibiting this axis could reduce patient immunity and eliminate antitumor immune functions, leading to potential clinical deterioration of δ-chemokines. However, analyses using the GSE24080 dataset did not confirm the correlation between XCR1 expression in MM cells and prognosis [18,19,20,21].

## 5. CX_3_CR1 and CX_3_CL1

The ligand for CX_3_C motif chemokine receptor 1 (CX_3_CR1) is CX_3_C motif chemokine ligand 1 (CX_3_CL1) [9], previously known as fractalkine [31]. CX3CR1 is expressed on NK cells, basophils, monocytes, macrophages, dendritic cells, CD8^+^ T cells, and activated CD4^+^CD45RO^+^ T cells [9,207]. CX_3_CL1 is initially synthesized as a membrane-attached protein that can act as an adhesion molecule for CX_3_CR1 [208,209]. Its extracellular portion can be cleaved and shed, producing soluble CX_3_CL1 [210]. Because CX_3_CL1 expression increases in activated endothelium, the CX_3_CL1–CX_3_CR1 axis directs leukocytes to inflammatory sites [208]. It also contributes to osteoclast differentiation [211]. In solid tumors, this axis helps the immune system eliminate cancer cells by promoting infiltration of CD8^+^ T cells and NK cells [212,213]. However, CX_3_CL1-CX_3_CR1 also exhibits protumor effects, such as recruiting M-MDSC [214] and TAM [215] to the tumor niche and inducing angiogenesis by acting on endothelial cells [216].

### CX_3_CR1 and CX_3_CL1 in MM

Lower CX_3_CL1 levels in otherwise healthy individuals have been associated with a higher risk of MM [105,106]. Since CX_3_CL1-CX_3_CR1 contributes to immune function, particularly via NK cells [209], reduced CX_3_CL1 may indicate decreased immune surveillance, permitting MGUS or MM to arise. In MM, serum CX_3_CL1 exceeds levels seen in healthy donors [90]. A similar pattern is observed in bone marrow plasma, where its concentration is higher in MM than in sMM and MGUS, correlating with ISS stage [217]. MM cells themselves typically do not produce large amounts of CX_3_CL1, but they induce endothelial cells to express more of it, partly via TNF-α [206]. CX_3_CL1 in the bone marrow correlates with tumor burden [217], indicating a connection to MM progression.

Certain MM lines express CX_3_CR1 [218], so only some patients’ MM cells may carry it. CX_3_CL1-CX_3_CR1 activation in MM cells increases their adhesion to fibronectin and vascular cell adhesion molecule-1 (VCAM-1) [218], which can lead to CAM-DR, and enhances osteoclastogenic factors. However, bone marrow levels of CX_3_CL1 in MM are not linked to bone disease [217], suggesting a limited role in myeloma bone disease.

Within the MM microenvironment, CX_3_CL1 levels in the bone marrow are positively associated with microvessel density [217], implying that it contributes to angiogenesis. Still, a bioinformatic analysis using KM-plotter (accessed 1 September 2024) and the GSE4204 [17,21] and GSE24080 datasets [18,19,20,21] indicates that CX_3_CL1-CX_3_CR1 is not critical overall for MM tumor processes, as neither CX_3_CL1 nor CX_3_CR1 expression in MM cells strongly correlates with prognosis (Table 6 and Table 7). Only in the CD2 and MS molecular subgroups does higher CX_3_CL1 predict better outcomes, suggesting antitumor potential in specific contexts.

## 6. Conclusions

Bioinformatic analysis indicates that CXCL17, CCR3, CCR5, and the CCL25–CCR9, CCL27/CCL28–CCR10, and XCL1–XCR1 axes correlate with poorer outcomes in MM patients, especially in the HY and LB molecular subgroups. These chemokines, receptors, and axes thus appear to play important roles in MM tumor processes. However, limited existing research does not fully explain how they drive malignancy. Their association with prognosis suggests they may be promising therapeutic targets. Possible interventions include receptor antagonists, receptor-blocking antibodies, or neutralizing antibodies against specific chemokines. It is important to note that these molecules not only influence MM but also help regulate immune function and may have additional roles unrelated to leukocytes. For this reason, the potential side effects of treatments targeting these proteins must be thoroughly assessed.

Another indication of bioinformatic analysis is the association of high expression of certain chemokines in MM cells with better patient prognosis. Specifically, these chemokines include CXCL16, CCL11, CCL17, CCL21, CCL27, and CX_3_CL1. These chemokines act on lymphocytes. These chemokines, or more precisely, the lymphocytes affected by these chemokines, participate in the immune and antitumor response. This demonstrates the dynamic nature of the interaction between MM cells and the immune system, as well as the immune system’s active fight against MM. This analysis examined patients with MM receiving standard treatment. A better understanding of this observed interaction will allow for a better understanding of the consequences of anticancer therapy. It will also facilitate the development of immunotherapies against MM, such as CAR T cell therapy. These chemokines may facilitate the migration of CAR T cells to the site of MM cell occupancy. These chemokines also act on other immune cells. These cells may cooperate with CAR T cells during therapy. However, the precise mechanisms remain to be explored. The observed correlations between rarely studied chemokines and tumor mechanisms in MM remain poorly understood. The limited information available and bioinformatic analyses indicate the significant potential of these chemokines in MM therapy. However, without significant interest from the scientific community, progress in MM treatment in this area will not be possible.

## 7. Limitations

It should be emphasized that the bioinformatics analysis used in this work has several limitations. Therefore, the data presented in our study should be analyzed with caution. The most important point is that the bioinformatics analysis is based on raw expression data from CD138+ MM cells. Therefore, the analysis only demonstrates the effects of chemokines on MM cells and the effects of MM cells on the MM bone marrow microenvironment via chemokines. However, it does not demonstrate the interactions of non-MM cells in the bone marrow of MM patients.

## Figures and Tables

**Table 1:** Association between the expression levels of less-studied α-chemokines and their receptors and survival in MM patients across molecular subgroups (from GSE4204 dataset) [17,21].

Chemokine or Receptor	In All Patients	CD1	CD2	PR	HY	LB	MF	MS	MY
Receptors									
CXCR5	-	↑	-	-	↓	↑	↓ *p* = 0.055	-	-
CXCR6	↑	↑	-	↑	-	↑ *p* = 0.094	↑ *p* = 0.065	-	↑ *p* = 0.078
GPR35	↑ *p* = 0.10	-	-	↑	↓ *p* = 0.052	↑ *p* = 0.089	-	-	-
Ligands									
CXCL13	-	↑	-	↑ *p* = 0.089	-	↓ *p* = 0.065	-	↓	-
CXCL14	-	↑ *p* = 0.082	↑ *p* = 0.090	↑	↓	-	↓ *p* = 0.068	-	-
CXCL16	↑	↑	↑	-	-	-	↑ *p* = 0.072	-	-
CXCL17	↓	↑	-	-	↓	↓	-	-	↓

Molecular subgroup of MM: CD1—MM with t(11;14) or t(6;14) (increased expression of CCND1 and CCND3, respectively). CD2—MM with t(11;14) or t(6;14) (increased expression of CCND1 and CCND3, respectively); expression of genes on chromosome 1p and early B-cell markers. PR—highly proliferative MM subgroup. HY—hyperdiploid molecular subgroup. LB—MM subgroup with a low number of bone lesions. MF—MM with t(14;16) or t(14;20) (increased expression of MAF and MAFB, respectively). MS—MM with t(4;14) (increased expression of FGFR3 and MMSET). MY—MM molecular subgroup with increased expression of MYC and BCL2L1 (Bcl-xL). ↑; blue background—higher expression is associated with better prognosis; ↓; red background—higher expression is associated with worse prognosis; gray background—no influence.

**Table 2:** Association between the expression levels of less-studied α-chemokines and their receptors and survival in MM patients across molecular subgroups (from GSE24080 dataset) [18,19,20,21].

Chemokine Receptor	Associated with Prognosis	Chemokine	Associated with Prognosis
CXCR5	-	CXCL13	-
		CXCL14	-
CXCR6	↑	CXCL16	↑
GPR35	-	CXCL17	-

↑; blue background—higher expression is associated with better prognosis; gray background—no influence.

**Table 3:** Association between the expression levels of β-chemokine receptors and survival in MM patients across molecular subgroups (from GSE4204 dataset) [17,21].

Receptor	In All Patients	CD1	CD2	PR	HY	LB	MF	MS	MY
CCR1	-	-	↑	↓ *p* = 0.085	↓	↓	-	-	↑ *p* = 0.063
CCR2	↑	-	-	-	↑	↓	-	-	↑
CCR3	↓	↑	-	-	↓	↓	↓ *p* = 0.058	-	-
CCR4	↓ *p* = 0.077	↓ *p* = 0.090	-	↑ *p* = 0.088	↓ *p* = 0.088	↓ *p* = 0.068	↓ *p* = 0.081	↑ *p* = 0.083	-
CCR5	-	↑ *p* = 0.069	↓	-	↓	↓	↑ *p* = 0.090	↑	-
CCR6	-	↓ *p* = 0.080	↑ *p* = 0.068	↓ *p* = 0.093	↓	↓ *p* = 0.098	↑	-	-
CCR7	↑	↓ *p* = 0.078	-	-	-	↓	↓ *p* = 0.055	-	↓
CCR8	-	-	-	-	↑	↓ *p* = 0.096	-	-	↑
CCR9	-	-	-	-	-	-	-	-	-
CCR10	↓	-	-	↓	↓ *p* = 0.072	↓	↓	↓	↑
PITPNM3	↑ *p* = 0.058	↑ *p* = 0.082	↑	↑	↑	-	↓	-	-

Molecular subgroup of MM: CD1—MM with t(11;14) or t(6;14) (increased expression of CCND1 and CCND3, respectively). CD2—MM with t(11;14) or t(6;14) (increased expression of CCND1 and CCND3, respectively), along with gene expression on chromosome 1p and expression of early B-cell markers. PR—highly proliferative MM subgroup. HY—hyperdiploid molecular subgroup. LB—MM subgroup with a low number of bone lesions. MF—MM with t(14;16) or t(14;20) (increased expression of MAF and MAFB, respectively). MS—MM with t(4;14) (increased expression of FGFR3 and MMSET). Y—MM molecular subgroup with increased expression of MYC and BCL2L1 (Bcl-xL). ↑; blue background—higher expression is associated with better prognosis; ↓; red background—higher expression is associated with worse prognosis; gray background—no influence.

**Table 4:** Association between β-chemokine expression levels and survival in MM patients across molecular subgroups (from GSE4204 dataset) [17,21].

Chemokine	In All Patients	CD1	CD2	PR	HY	LB	MF	MS	MY
CCL1	↑	-	-	-	-	↑	↑	↑	↑
CCL2	↑	-	↑ *p* = 0.094	-	-	-	↑	↑ *p* = 0.070	-
CCL3	-	↑	↑	-	-	↓	-	↑ *p* = 0.095	-
CCL4	-	-	↑ *p* = 0.072	-	-	↑ *p* = 0.052	-	↑	-
CCL5	-	-	-	-	↑ *p* = 0.070	↑	↓	-	↓ *p* = 0.098
CCL7	-	-	-	↓	↑	-	-	-	↓
CCL8	↑	-	-	↑	↑	↑ *p* = 0.10	↑	↓	↑ *p* = 0.073
CCL11	↑	↑	-	↑ *p* = 0.078	↓	-	-	-	-
CCL13	-	-	-	-	-	↓ *p* = 0.057	↓	↓ *p* = 0.061	-
CCL14	-	↓	-	↑	↓	↓	-	-	-
CCL14-CCL15	-	-	↑ *p* = 0.058	↑	↓	-	↑	↑	-
CCL16	-	↑	↓ *p* = 0.054	↑ *p* = 0.098	-	-	-	↓ *p* = 0.099	↑ *p* = 0.052
CCL17	↑	↑ *p* = 0.083	↑	-	↑	-	-	-	-
CCL18	↑	-	-	-	↑	↓	-	↑ *p* = 0.066	↑
CCL19	-	-	-	-	-	-	-	↓	↑ *p* = 0.066
CCL20	↑ *p* = 0.091	↓ *p* = 0.10	↓ *p* = 0.069	↑	-	↓ *p* = 0.10	-	↑	-
CCL21	↑	↑	↑	-	↑	↑	↑ *p* = 0.058	-	-
CCL22	-	-	-	-	↓ *p* = 0.088	-	↓ *p* = 0.073	-	-
CCL23	↑	-	-	-	-	↑ *p* = 0.097	↑ *p* = 0.056	↑ *p* = 0.081	-
CCL24	↑	-	-	-	-	-	-	↑	↑ *p* = 0.052
CCL25	-	↑	↓ *p* = 0.10	↓	-	↓	↓	↓ *p* = 0.074	↓
CCL26	-	-	-	-	-	-	↓	-	↓
CCL27	↑	↑ *p* = 0.053	-	↑	↓ *p* = 0.10	↓ *p* = 0.076	↑	-	-
CCL28	↓ *p* = 0.097	-	-	-	↓ *p* = 0.063	↓	↑	-	-

Molecular 1.—MM with *t*(11;14) or *t*(6;14) (increased expression of *CCND1* and *CCND3*, respectively). CD2—MM with *t*(11;14) or *t*(6;14) (increased expression of *CCND1* and *CCND3*, respectively), along with gene expression on chromosome 1p and early *B*-cell marker expression. PR—highly proliferative MM subgroup. HY—hyperdiploid molecular subgroup. LB—MM subgroup with a low number of bone lesions. MF—MM with *t*(14;16) or *t*(14;20) (increased expression of *MAF* and *MAFB*, respectively). MS—MM with *t*(4;14) (increased expression of *FGFR3* and *MMSET*). MY—MM molecular subgroup with increased expression of *MYC* and *BCL2L1* (*Bcl-xL*). ↑; blue background—higher expression is associated with better prognosis; ↓; red background—higher expression is associated with worse prognosis; gray background—no influence.

**Table 5:** Association between the expression levels of less-studied β-chemokines and their receptors and survival in MM patients across molecular subgroups (from GSE24080 dataset) [18,19,20,21].

Chemokine Receptor	Associated with Prognosis And	Chemokine	Associated with Prognosis And	Chemokine	Associated with Prognosis And
CCR1	-	CCL3	-		
CCR2	↑ *p* = 0.081	CCL2	↑ *p* = 0.087	CCL7	-
		CCL8	↑	CCL13	-
CCR3	↓ *p* = 0.084	CCL11	-	CCL23	-
		CCL24	↑ *p* = 0.066	CCL26	-
		CCL14-CCL15	↑ *p* = 0.1		
CCR4	-	CCL17	↑ *p* = 0.074	CCL22	-
CCR5	-	CCL4	-	CCL5	-
		CCL16	-		
CCR6	-	CCL20	-		
CCR7	-	CCL19	-	CCL21	↑
CCR8	-	CCL1	↑		
PITPNM3	↑ *p* = 0.088	CCL18	↑		
CCR9	↓ *p* = 0.085	CCL25	-		
CCR10	↓ *p* = 0.085	CCL27	-	CCL28	↓

↑; blue background—higher expression is associated with better prognosis; ↓; red background—higher expression is associated with worse prognosis; gray background—no influence.

**Table 6:** Association between the expression levels of γ- and δ-chemokines and their receptors and survival in MM patients across molecular subgroups (from GSE4204 dataset) [17,21].

Chemokine or Receptor	In All Patients	CD1	CD2	PR	HY	LB	MF	MS	MY
γ-chemokines									
XCL1	-	-	-	↓ *p* = 0.072	↓	↓ *p* = 0.094	-	-	-
XCL2	N/A	N/A	N/A	N/A	N/A	N/A	N/A	N/A	N/A
XCR1	↓	↑	↓	↓	↓	-	-	-	↑
δ-chemokines									
CX_3_CL1	-	-	↑	-	-	-	↑ *p* = 0.056	↑	-
CX_3_CR1	-	↑ *p* = 0.059	-	-	-	-	-	-	-

Molecular subgroup of MM: CD1—MM with *t*(11;14) or *t*(6;14) (increased expression of *CCND1* and *CCND3*, respectively). CD2—MM with *t*(11;14) or *t*(6;14) (increased expression of *CCND1* and *CCND3*, respectively), along with gene expression on chromosome 1p and early *B*-cell marker expression. PR—highly proliferative MM subgroup. HY—hyperdiploid molecular subgroup. LB—MM subgroup with a low number of bone lesions. MF—MM with *t*(14;16) or *t*(14;20) (increased expression of *MAF* and *MAFB*, respectively). MS—MM with *t*(4;14) (increased expression of *FGFR3* and *MMSET*). MY—MM molecular subgroup with increased expression of *MYC* and *BCL2L1* (*Bcl-xL*). ↑; blue background—higher expression is associated with better prognosis; ↓; red background—higher expression is associated with worse prognosis; gray background—no influence.

**Table 7:** Association between the expression levels of γ- and δ-chemokines and their receptors and survival in MM patients across molecular subgroups (from GSE24080 dataset) [18,19,20,21].

Chemokine Receptor	Influence on Prognosis		Chemokine
XCR1	-	XCL1	-
		XCL2	-
CX_3_CR1	-	CX_3_CL1	-

Gray background—no influence on prognosis.

## Data Availability

No new data were created or analyzed in this study. Data sharing is not applicable to this article.
